# MITF-Mediated lncRNA CCDC183-As1 Promotes the Tumorigenic Properties and Aerobic Glycolysis of Bladder Cancer via Upregulating TCF7L2

**DOI:** 10.1155/2022/6785956

**Published:** 2022-07-31

**Authors:** Wei Cai, Meng Wei, Zhen Su

**Affiliations:** ^1^Department of Orthopedics, The Affiliated Huai'an No. 1 People's Hospital of Nanjing Medical University, Huai'an, Jiangsu, China; ^2^Department of Anesthesiology, The Affiliated Huai'an No. 1 People's Hospital of Nanjing Medical University, Huai'an, Jiangsu, China

## Abstract

As a primary malignancy tumor of the urology system, bladder cancer (BC) is characterized by its high recurrence and metastasis characteristics. Despite the great improvement in clinical interventions over the past decades, the outcomes of BC patients are still unsatisfactory. Novel molecular mechanisms for developing effective diagnostic and therapeutic strategies are urgently needed; therefore, we screened the lncRNA expression profile in four pairs of BC tissues, showing that CCDC183-AS1 was the most upregulated lncRNA. Subsequently, results of CCK-8, EdU, Transwell, and aerobic glycolysis detection showed that CCDC183-AS1 plays an oncogene role in BC progression. Furthermore, an investigation of the downstream and upstream factors of CCDC183-AS1 identified a novel MITF/CCDC183-AS1/miR-4731-5p/TCF7L2 axis in BC progression, which might furnish novel insights for developing effective diagnostic and therapeutic strategies for BC.

## 1. Introduction

Bladder cancer (BC) ranks the second most common cancer in the urology system with more than 550,000 newly diagnosed and 20,000 BC-related deaths every year [1]. The most prominent features of BC are the high recurrence rate and local or distant metastasis, contributing to the poor prognosis and outcomes of BC [2]. Despite the great improvement in clinical treatment including radiation, surgery, and chemotherapy, the 5-year survival rate is still poor [3]. Hence, identifying novel mechanisms of BC for developing effective diagnosis and treatment strategies is demanded.

Long noncoding RNA (lncRNA) is a type of RNA more than 200 nucleotides long without protein-coding capability [4]. lncRNA exerts its role via four mechanisms including decoys, guides, molecular scaffolds, and signaling molecules to regulate the transcription or expression of genes [5]. Currently, the molecular and biological functions of lncRNAs in various diseases have been well documented, such as cardiovascular diseases, neuropathic pain, diabetes, and human cancers [6–9]. Accumulating evidence suggests that dysregulation of lncRNAs participates in the development of BC. Chen et al. revealed that lncRNA LNMAT contributes to the lymphatic metastasis of BC [10]. He et al. demonstrated that lncRNA BLACAT2 aggravates BC-associated lymphangiogenesis and lymphatic metastasis [11]. Luo et al. found that lncRNA PR11-89 modulates tumorigenesis and ferroptosis resistance of BC through sponging miR-129-5p [12]. Tan J et al. demonstrated a novel lncRNA TUG1/miR-320a/FOXQ1 pathway in BC progression [13].

This study explored a new functional lncRNA in the progression of BC. First, we collected four pairs of BC tumor samples and comparative normal samples for the lncRNA microarray experiment, showing that lncRNA coiled-coil domain-containing 183 antisense RNA 1 (CCDC183-AS1) was markedly increased in BC tumor samples compared to normal samples. Next, the biological functions of CCDC183-AS1 in BC cells were investigated, showing that CCDC183-AS1 knockdown retarded cell viability, migration, and invasion. Furthermore, CCDC183-AS1 was involved in the modulation of aerobic glycolysis. Subsequently, our study demonstrated the molecular mechanisms including the downstream and upstream regulators of CCDC183-AS1, identifying a novel MITF/CCDC183-AS1/miR-4731-5p/TCF7L2 axis in BC progression, which may be useful for developing new diagnostic or treatment strategies for BC.

## 2. Materials and Methods

### 2.1. Tissue Collection

All thirty paired BC samples and adjacent normal samples were collected from patients diagnosed with BC who received surgery at The Affiliated Huai'an NO. 1 People's Hospital of Nanjing Medical University (2019–2021). The Ethics Committee of The Affiliated Huai'an NO. 1 People's Hospital of Nanjing Medical University approved this study. Informed consents were obtained, and two pathologists confirmed the BC diagnosis.

### 2.2. lncRNA Microarray Analysis

Total RNA isolated from four pairs of BC tumor samples and normal samples was analyzed with RT2 lncRNA PCR Arrays (QIAGEN NV Corporate, Venlo, The Netherlands) and the Data Analysis Center as before [13]. The experiment was repeated in triplicate (fold change > 1.5 and *P* values < 0.05).

### 2.3. Cell Culturing and Transfection

Cell lines (SW780, UMUC3, T24, HT1367, and 5637) were procured from the Shanghai Institute of Cell Biology (Shanghai, China) and the bladder cell (SV-HUC-1) was procured from the ATCC. Cells were cultured using RPMI-1640 medium (Invitrogen, Carlsbad, CA, United States) in a humidified 5% CO [2] environment at 37°C. siRNAs, mimics, and inhibitors were synthesized and commercially obtained from Gene-Pharma (Shanghai, China). All transfections were performed using a Lipofectamine 3000 kit (Thermo Fisher Scientific, Carlsbad, CA, USA).

### 2.4. RNA Extraction and Quantitative Real-Time PCR (qRT-PCR)

RNAs were harvested when cells were 80% confluent. RNAs were harvested by an RNA extraction kit (Invitrogen) and reverse-transcribed into cDNA. PCR amplicons were detected using a SyBr Green PCR system. The primers information was described as follows: CCDC183-AS1, F : GACTTGATCCGTTGGCCTGA, R : CTTGGACTTCCCCTCGAACC, miR-4731-5p, fF : GGGGGCCACATGAGT, R : GGTCCAGTTTTTTTTTTTTTTTCACA, TCF7L2, F : CCGCCCGAACCTCTAACAAA, R : TCAGTCTGTGACTTGGCGTC, U6, F : CTCGCTTCGGCAGCACA, R : ACGCTTCACGAATTTGCGT, GAPDH, F : CACCATTGGCAATGAGCGGTTC, R : AGGTCTTTGCGGATGTCCACGT. The data analysis was conducted using the 2–∆∆Ct method.

### 2.5. Western Blotting

Western blot analysis was carried out with the primary antibody TCF7L2 (ab134275, 1 : 500) and HPR-labeled secondary antibody (ab205718, 1 : 20000). In brief, total proteins were isolated using a radioimmunoprecipitation assay (Beyotime, Jiangsu, China) and quantified using a bicinchoninic acid assay. The proteins were separated using 12% sodium dodecylsulphate-polyacrylamide gel electrophoresis (Beyotime) and then transferred to polyvinylidene fluoride membranes (Millipore, Burlington, MA, USA). 5% milk was used to block membranes, then incubated with TCF7L2 antibody for 12 hours at 4°C, followed by HPR-labeled antibody for 2 hours. An enhanced chemiluminescence system (Millipore) was used to capture the signals.

### 2.6. Subcellular Fractionation

The determination of subcellular distribution was conducted using a PARIS Kit (Life Technologies, Pudong, Shanghai, China) following the manufacturer's guides.

### 2.7. Cell Proliferation Assay

A CCK-8 (Beyotime, Shanghai, China) was applied to measure cell proliferation ability. The cells were cultured in a 96-well plate at 37°C for 24 h before the addition of CCK-8 solution for 1 hour. Cell absorbance was analyzed using a microplate reader (Potenov, Beijing, China) at 450 nm wavelength to calculate cell growth.

### 2.8. Ethynyl-2′-Deoxyuridine (EdU)

A BeyoClick™ EdU Kit (C0075S, Beyotime Biotechnology, Shanghai, China) was used to perform the EdU assay following the manufacturer's guides. Cells were plated in a 12-well chamber (Ibidi, Germany) and supplied with 200 *μ*l EdU (50 *μ*M) for 2 h incubation. Subsequently, cells were added with 4% paraformaldehyde for 15 min and permeabilized with 0.3% Triton X-100 for 10 min and then labeled with 5 *μ*g/ml of Hoechst 33342 for 30 min. The images were obtained by a confocal microscope (LSM 510, META laser scanning microscope, Zeiss).

### 2.9. Detection of Cell Migration and Invasion

Cells (10^6^ cells/mL, 200 *μ*L) were seeded in an 8-*μ*m Transwell chamber (Corning, NY, USA). The chamber (lower one) was filled with cells or 10% FBS medium (600 *μ*L). After 24 hours, the cells were added with 4% paraformaldehyde and stained for 20 min with 0.5% crystal violet staining solution (Sigma-Aldrich). Subsequently, the cells in the chamber (upper one) were removed, and then migrated cells were recorded using an inverted fluorescence microscope (TE2000, Nikon, Japan). The chamber added with Matrigel (BD Biosciences, CA, USA) was for invasion detection.

### 2.10. Cellular Glycolysis and Oxidative Phosphorylation Detection

The rate of extracellular acidification (ECAR) was used to detect glycolysis level, and the OCR was used to detect oxidative phosphorylation level using the XF96 metabolic flux analyzer (Seahorse Biosciences, Billerica, MA, USA) as a previous study [14].

### 2.11. Measurement of Glucose Uptake, Lactate Production, and ATP Level

Glucose-free DMEM was used to culture BC cells (16 hours) and replaced with high-glucose DMEM (24 hours). A PicoProbeTM Glucose Fluorometric Assay Kit (K688, BioVision, United States) was used to detect intracellular glucose levels. The lactate levels were determined in the culture medium by a PicoProbeTM Lactate Fluorometric Assay Kit (K688, BioVision, United States). Furthermore, the cellular ATP level was measured by an ATP assay kit (Promega, Madison, WI). Experiments were performed in triplicate.

### 2.12. RNA Pull-Down Assay

CCDC183-AS1 and miR-4731-5p were labeled with T7 Biotin by the T7 Enzyme mix and Biotin RNA Labeling Mix (RiboBio, Guangzhou, China), purified by a RNA Clean and Concentrator™-5 kit (Zymo Research, Orange County, CA, USA). After sonication, the cell extracts were added with Dynabeads M-280 Streptavidin (Invitrogen), which biotinylated probes were precoupled. TRIzol reagent (Invitrogen) was used to lyse the complexes.

### 2.13. Dual-Luciferase Reporter Gene Assay

CCDC183-AS1 and TCF7L2 3ʹUTR sequences harboring miR-4731-5p binding sites were planted into pmirGLO vectors generating wild-type (WT) CCDC183-AS1 and TCF7L2. The mutant-type (MUT) vectors were obtained using mutant miR-4731-5p binding sites. After transfection, the luciferase activity was measured using a microplate reader.

### 2.14. Statistical Analysis

GraphPad Prism 6.0 (GraphPad Software, San Diego, CA, USA) and SPSS 19 were used to conduct statistical analysis. For data analysis, Student's *t*-tests (two-tailed) and one-way ANOVA were conducted. Experimental data are shown as the mean ± SD. *P* < 0.05 was treated as statistically significant.

## 3. Results

### 3.1. CCDC183-As1 Is Highly Expressed in BC

**Figure 1:**
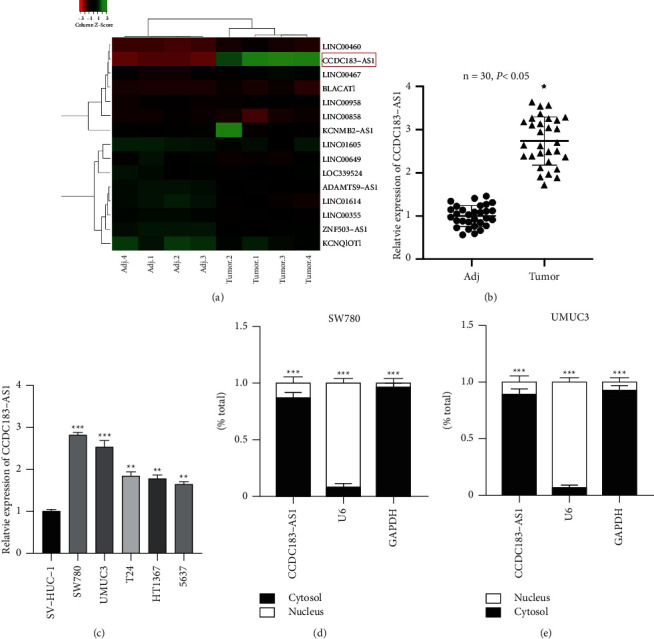
CCDC183-AS1 is highly expressed in BC. (a) Results of microarray analysis of four pairs of BC samples. (b) CCDC183-AS1 expression in thirty pairs of BC tissues was determined using qRT-PCR. (c) CCDC183-AS1 expression in BC cells was determined using qRT-PCR. (d, e) Cellular distribution of CCDC183-AS1 in BC cells was detected by cellular fragment assay.

CCDC183-AS1 was markedly increased in BC tumor samples compared to the adjacent normal samples (Figure 1(a)), and this was confirmed by the data from thirty pairs of BC tissues examined by qRT-PCR (Figure 1(b)). In the BC cell lines (SW780, UMUC3, T24, HT1367, and 5637), CCDC183-AS1 expression was higher compared to the bladder cells (SV-HUC-1) (Figure 1(c)). Intracellular distribution results have shown that CCDC183-AS1 was mainly located in the cell cytosol (Figures 1(d) and 1(e)). CCDC183-AS1 might play a critical role in BC pathology.

### 3.2. CCDC183-As1 Aggravates BC Tumorigenic Properties

**Figure 2:**
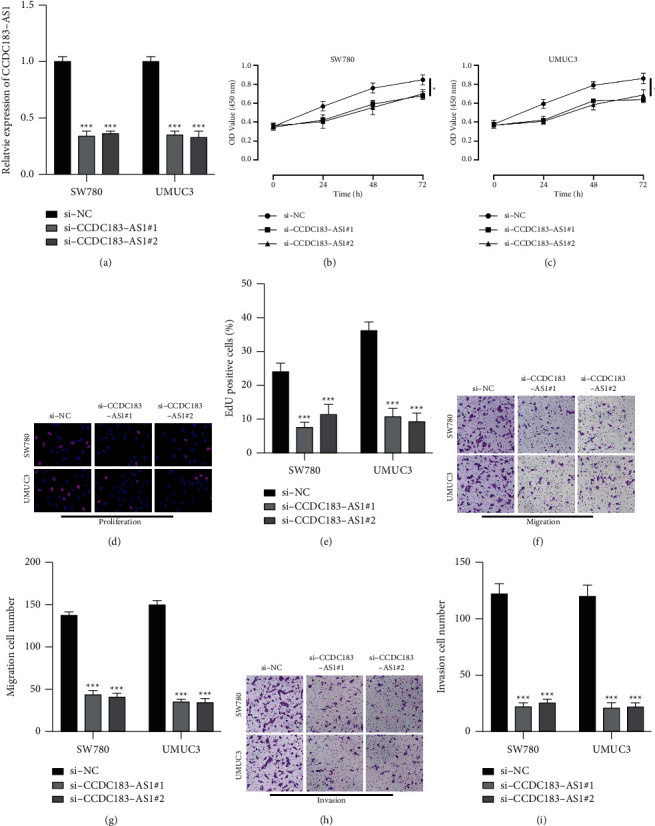
CCDC183-AS1 aggravates BC tumorigenic properties. (a) CCDC183-AS1 expression in UMUC3 and SW780 cells pretransfected with si-NC, si-CCDC183-AS1#1, and si-CCDC183-AS1#2 was determined using RT-qPCR. (b, c) CCK-8 and (d, e) EdU experiments were conducted to detect cell proliferation in UMUC3 and SW780 cells. (f, g) The Transwell migration experiment was conducted to measure cell migration in UMUC3 and SW780 cells. (h, i) The Transwell invasion experiment was performed to detect cell migration in UMUC3 and SW780 cells.

We stably transfected the siRNA targeting CCDC183-AS1 into UMUC3 and SW780 cells (Figure 2(a)). Results of the CCK-8 assay (Figures 2(b) and 2(c)) and EdU assay (Figures 2(d) and 2(e)) demonstrated that CCDC183-AS1 knockdown retarded BC cell proliferation. Subsequently, Transwell assay results suggested that downregulated CCDC183-AS1 attenuated cell migration (Figures 2(f) and 2(g)) and invasion (Figures 2(h) and 2(i)), indicating that CCDC183-AS1 might function as an oncogene role in BC development.

### 3.3. Target Relationship between CCDC183-As1 and miR-4731-5p

**Figure 3:**
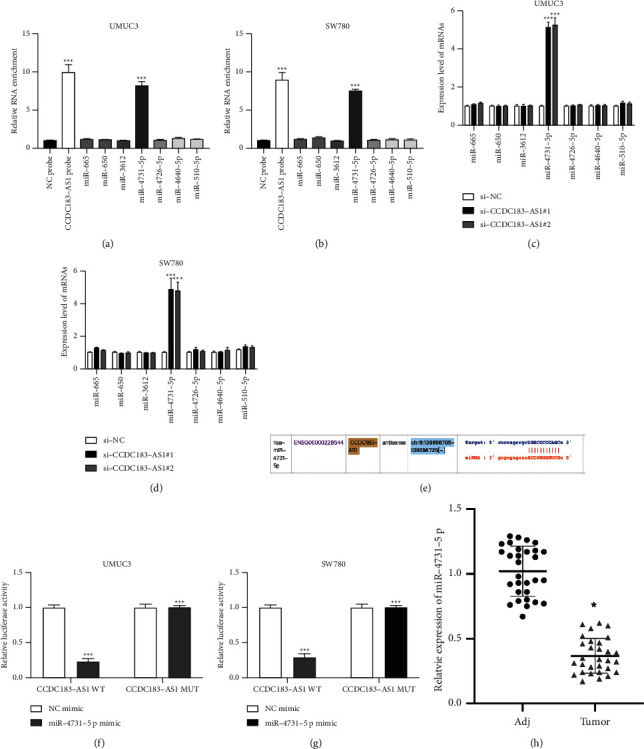
CCDC183-AS1 sponges miR-4731-5p. (a, b) The evaluation of putative miRNA enrichments in the complex of NC probes and CCDC183-AS1 probes in UMUC3 and SW780 cells was determined using a biotinylated RNA pull-down assay. (c, d) RT-PCR was conducted to measure the expression of putative miRNA expressions in CCDC183-AS1 downregulated UMUC3 and SW780 cells were measured by RT-PCR. (e) Putative binding sites of miR-4731-5p on CCDC183-AS1. (f, g) The binding between CCDC183-AS1 and miR-4731-5p in UMUC3 and SW780 was determined by dual-luciferase reporter gene assay. (h) Relative expression of miR-4731-5p in thirty pairs of BC tissues was detected by qRT-PCR.

lncRNAs act through sponging miRNAs and regulating gene expression has been well investigated [15]. Seven putative miRNAs were predicted as the target of CCDC183-AS1 by the ENCORI (https://starbase.sysu.edu.cn/index.php) database [16] (CLIP Data-high stringency (≥ 3)). The biotinylated RNA pull-down assay showed that miR-4731-5p was significantly enriched in CCDC183-AS1 probe complexes compared to other putative miRNAs, indicating that CCDC193-AS1 might sponge miR-4731-5p (Figures 3(a) and 3(b)). Furthermore, miR-4731-5p expression in UMUC3 and SW780 cells was negatively regulated by CCDC183-AS1 (Figures 3(c) and 3(d)). The putative target sites of miR-4731-5p on CCDC183-AS1 are presented in Figure 3(e) and the dual-luciferase assay confirmed that CCDC183-AS1 could sponge miR-4731-5p in UMUC3 and SW780 cells (Figures 3(f) and 3(g)). RT-PCR results suggested that miR-4731-5p expression in BC tumor samples were downregulated compared to adjacent normal samples (Figure 3(h)).

### 3.4. MiR-4731-5p Retarded BC Tumorigenic Properties

**Figure 4:**
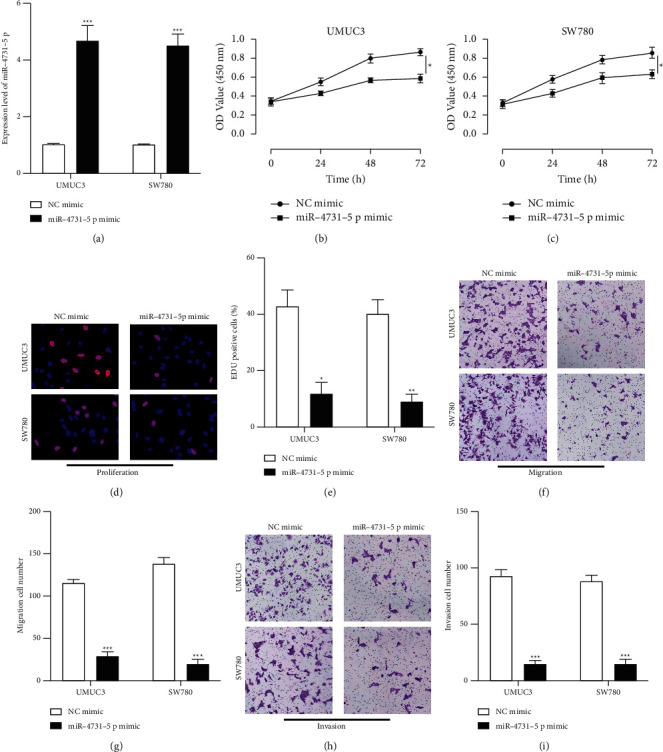
miR-4731-5p retards BC tumorigenic properties. (a) miR-4731-5p expression in UMUC3 and SW780 cells pretransfected with NC mimic and miR-4731-5p mimic was measured by RT-PCR. (b, c) CCK-8 assay and (d, e) EdU assay were conducted to measure cell proliferation in UMUC3 and SW780 cells. (f, g) The Transwell migration experiment was conducted to measure cell migration in UMUC3 and SW780 cells. (h, i) Cell invasion ability was determined by the Transwell invasion assay.

To understand the functional role of miR-4731-5p in BC, we stably infected miR-4731-5p mimics and its normal into UMUC3 and SW780 cells (Figures 4(b) and 4(c)) and EdU (Figures 4(d) and 4(e)) assays found that miR-4731-5p overexpression markedly repressed cell proliferation. Moreover, the results of the Transwell assay indicated that overexpressed miR-4731-5p markedly decreased cell migration ability (Figures 4(f) and 4(g)) and invasion ability (Figures 4(h) and 4(i)).

### 3.5. Target Relationship between miR-4731-5p and TCF7L2

**Figure 5:**
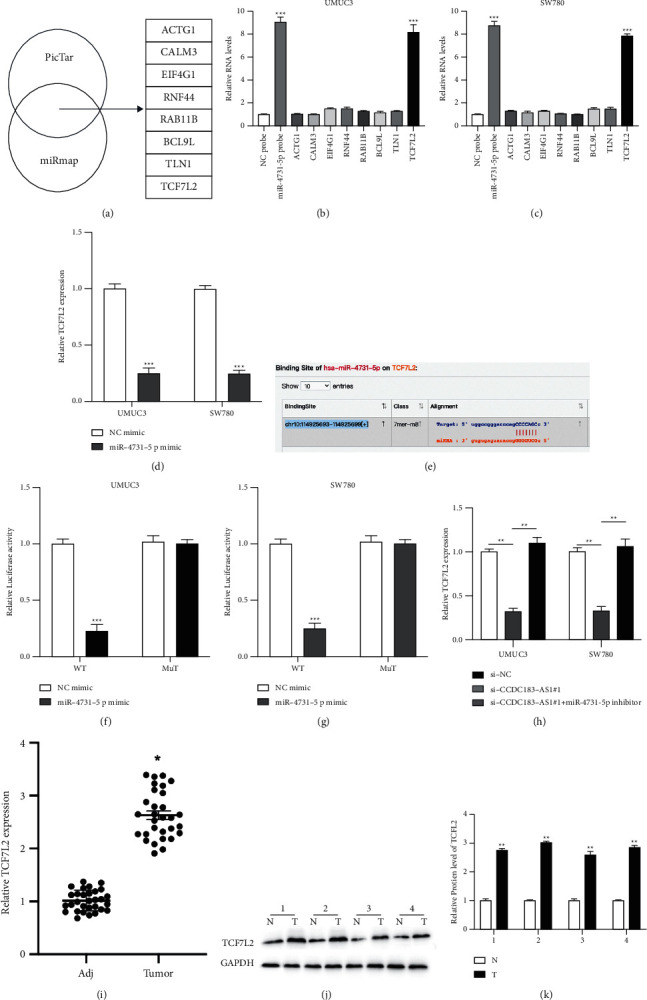
Target relationship between miR-4731-5p and TCF7L2. (a) Results of bioinformatics analysis. (b, c) The evaluation of putative mRNA enrichments in the complex of NC probes and miR-4731-5p probes in UMUC3 and SW780 cells was determined using a biotinylated RNA pull-down assay. (d) RT-PCR assay was conducted to measure TCF7L2 miRNA expressions in miR-4731-5p overexpressed UMUC3 and SW780 cells. (e) Putative binding sites of miR-4731-5p on TCF7L2. (f, g) The binding between TCF7L2 and miR-4731-5p was evaluated by a dual-luciferase reporter gene assay. (h) TCF7L2 expression in UMUC3 and SW780 cells preinfected with si-NC, si-CCDC183-AS1#1, and si-CCDC183-AS1#1 + miR-4731-5p inhibitor was measured by RT-PCR. (i) Relative expression of TCF7L2 in thirty pairs of BC tissues was detected by qRT-PCR. (j, k) TCF7L2 expression in four pairs of BC tissues was detected by western blotting.

Bioinformatics analysis (PicTar: https://pictar.mdc-berlin.de/and miRmap: https://mirmap.ezlab.org/) (CLIP Data-high stringency ≥ 5, Degradome Data-high stringency ≥ 3, and AgoExpNum ≥ 7) was conducted (Figure 5(a)). Biotinylated RNA pull-down combined with RT-PCR assays suggests that miR-4731-5p might target Transcription Factor 7 Like 2 (TCF7L2) in cells (Figures 5(b) and 5(c)). Besides, miR-4731-5p markedly reduced TCF7L2 expression in BC cells (Figure 5(d)). The putative target sites of miR-4731-5p on TCF7L2 are presented in Figure 5(e). The association between miR-4731-5p and TCF7L2 was confirmed by a dual-luciferase gene assay (Figures 5(f) and 5(g)). TCF7L2 expression in UMUC3 and SW780 cells was reduced by CCDC183-AS1 siRNA and increased by the miR-4731-5p inhibitor (Figure 5(h)). Furthermore, RT-PCR results showed that TCF7L2 expression in BC tumor samples was significantly upregulated compared to adjacent samples (Figure 5(i)), and these results were confirmed by western blotting of four pairs of BC tissues (Figures 5(j) and 5(k)).

### 3.6. CCDC183-As1 Promotes BC Tumorigenic Properties via miR-4731-5p/TCF7L2

**Figure 6:**
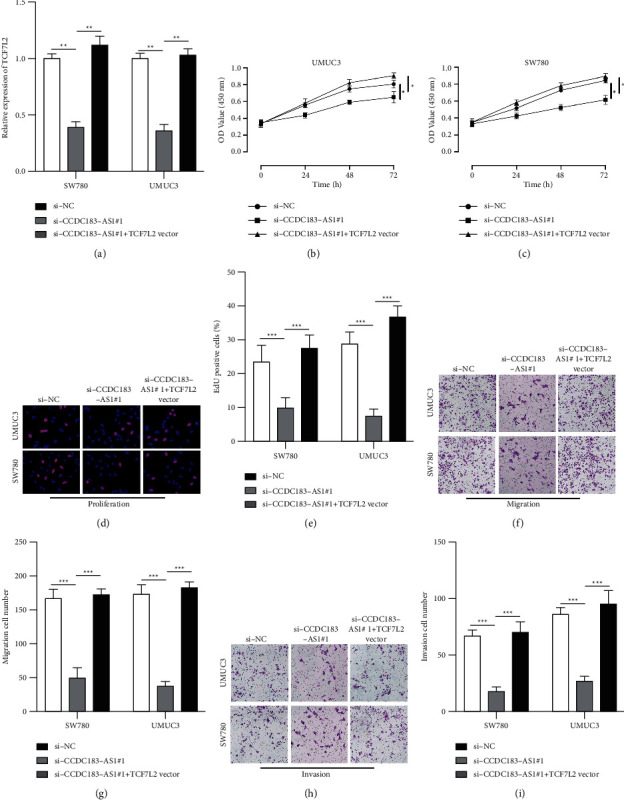
CCDC183-AS1 promotes BC tumorigenic properties via miR-4731-5p/TCF7L2. (a) TCF7L2 expression in UMUC3 and SW780 cells pretransfected with si-NC, si-CCDC183-AS1#1, and si-CCDC183-AS1#1 + TCF7L2 vector was measured by RT-PCR. (b-c) CCK-8 assay and (d, e) EdU assay were conducted to measure cell proliferation in UMUC3 and SW780 cells. (f, g) The Transwell migration experiment was conducted to measure the migration of UMUC3 and SW780 cells. (h, i) The Transwell invasion experiment was performed to detect the migration of UMUC3 and SW780 cells.

To understand whether CCDC183-AS1 exerts its biological function through TCF7L2, the si-NC, si-CCDC183-AS1#1, and si-CCDC183-AS1#1+TCF7L2 vectors were transfected into UMUC3 and SW780 cells, showing that CCDC183-AS1 knockdown reduced TCF7L2 expression in UMUC3 and SW780 cells, and TCF7L2 vector reversed this trend (Figure 6(a)). CCK-8 assay (Figures 6(b) and 6(c)) and EdU assay (Figures 6(d) and 6(e)) suggested that TCF7L2 overexpression had an opposite effect to downregulated CCDC183-AS1 on cell proliferation. Results of Transwell assay results indicated the same phenomena on cell migration ability (Figures 6(f) and 6(g)) and invasion ability (Figures 6(h) and 6(i)). In summary, sufficient evidence confirmed that CCDC183-AS1 modulated TCF7L2 expression, thus affecting BC cellular functions.

### 3.7. CCDC183-As1 Modulates Aerobic Glycolysis in BC via Upregulating TCF7L2

**Figure 7:**
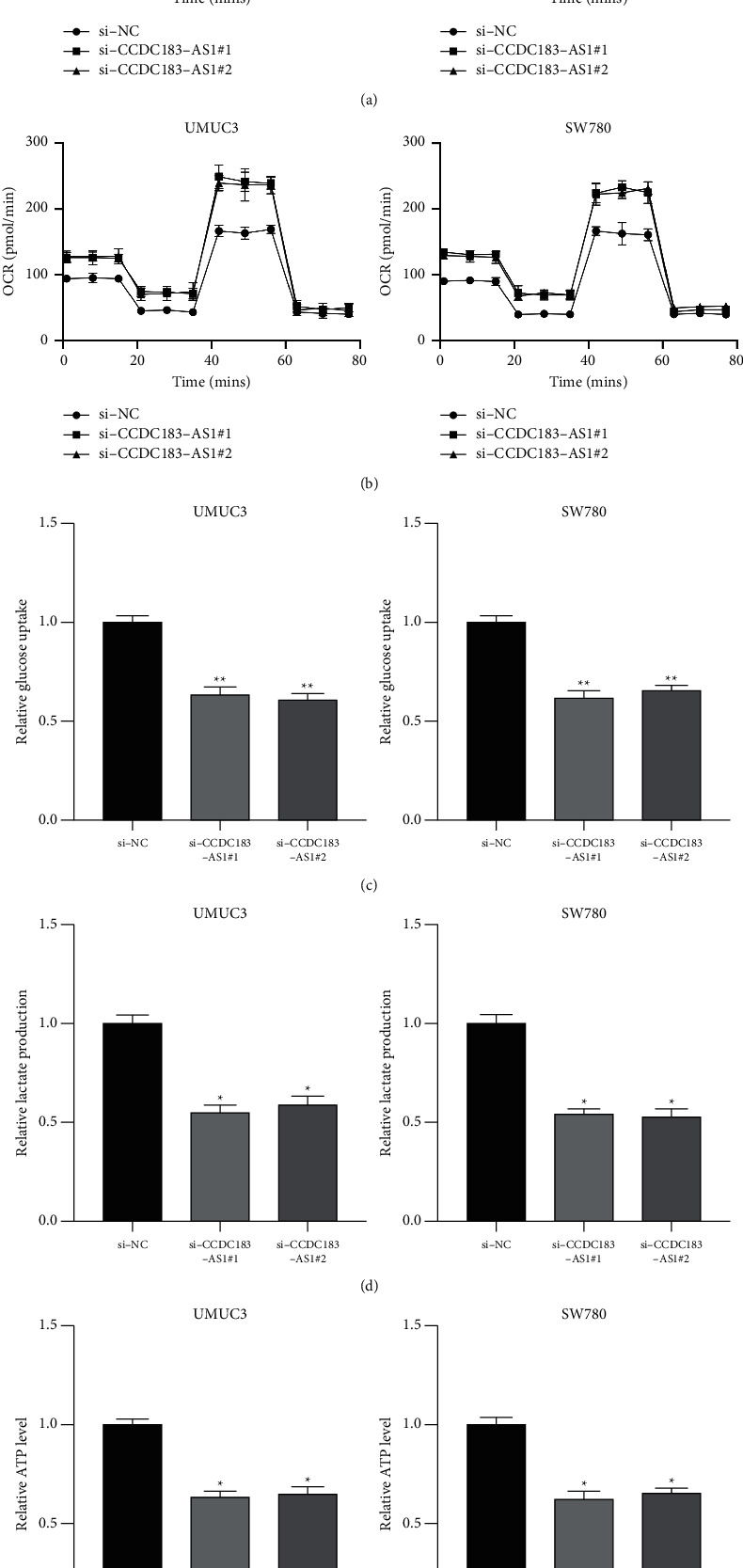
CCDC183-AS1 modulates aerobic glycolysis in BC cells. (a) ECAR of UMUC3 or SW780 cells preinfected with si-NC, si-CCDC183-AS1#1, and si-CCDC183-AS1#2. (b) O2 consumption rate (OCR) of UMUC3 or SW780 cells preinfected with si-NC, si-CCDC183-AS1#1, and si-CCDC183-AS1#2. (c) Glucose uptake was determined in UMUC3 or SW780 cells preinfected with si-NC, si-CCDC183-AS1#1, and si-CCDC183-AS1#2. (d) Lactate production in UMUC3 or SW780 cells preinfected with si-NC, si-CCDC183-AS1#1, and si-CCDC183-AS1#2. (e) ATP level was determined in UMUC3 or SW780 cells pretransfected with si-NC, si-CCDC183-AS1#1, and si-CCDC183-AS1#2.

Given the essential role of TCF7L2 in aerobic glycolysis [17,18], and the relationship between CCDC183-AS1 and TCF7L2. We investigated the function of CCDC183-AS1 on aerobic glycolysis. As shown in Figure 7(a), the glycolytic process (ECAR) markedly reduced in CCDC183-AS1 downregulated UMUC3 and SW780 cells. Results of mitochondrial respiration (OCR) suggested that OCR increased significantly in CCDC183-AS1 downregulated UMUC3 and SW780 cells (Figure 7(b)). Furthermore, CCDC183-AS1 knockdown had negative effects on glucose uptake (Figure 7(c)), lactate production (Figure 7(d)), and ATP level (Figure 7(e)) in UMUC3 and SW780 cells, indicating that CCDC183-AS1 exerts its promotive effect on aerobic glycolysis.

**Figure 8:**
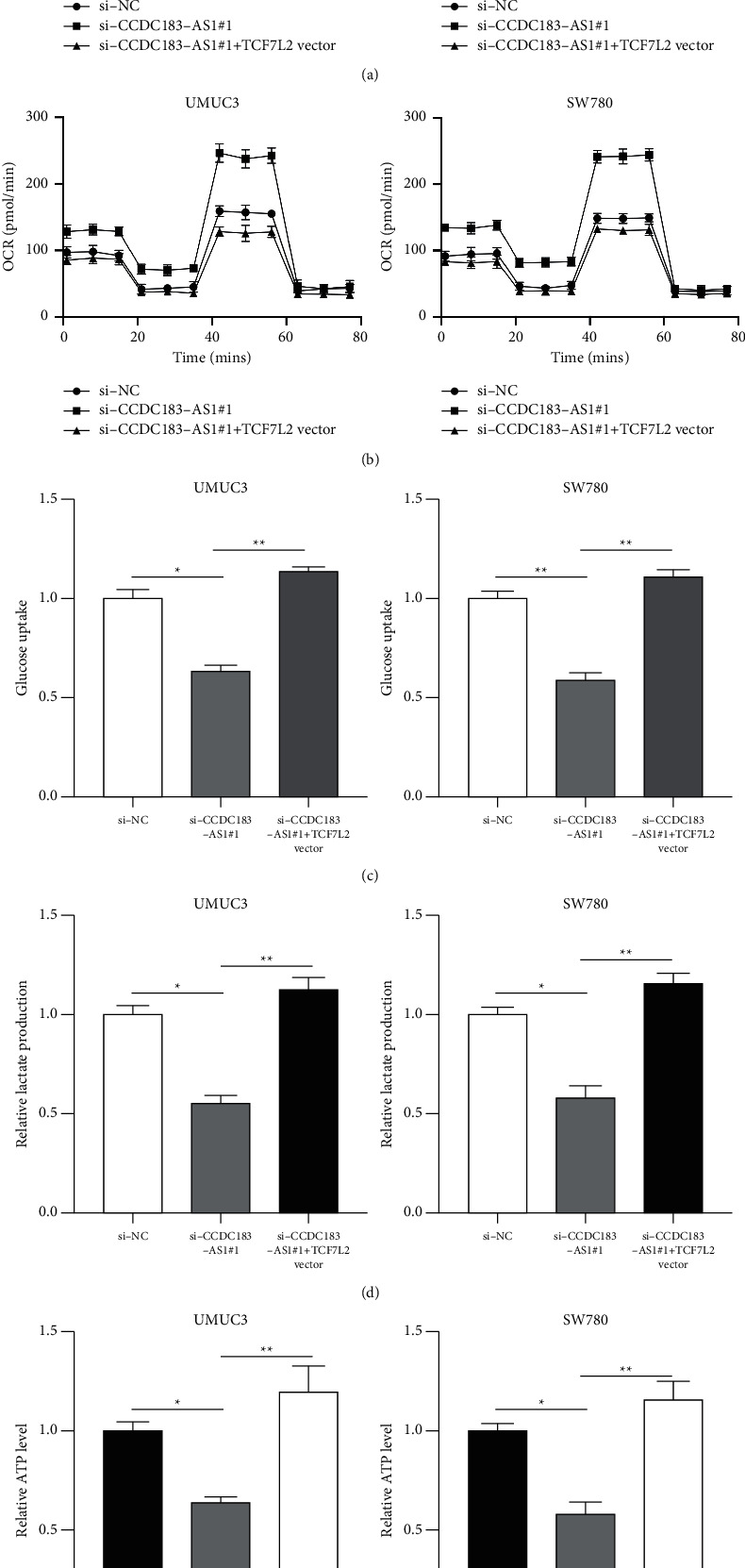
TCF7L2 is essential for the functional role of CCDC183-AS1 in modulating aerobic glycolysis in BC. (a) ECAR of UMUC3 or SW780 cells preinfected with si-NC, si-CCDC183-AS1#1, and si-CCDC183-AS1#1 + TCF7L2 vector. (b) OCR of UMUC3 or SW780 cells preinfected with si-NC, si-CCDC183-AS1#1, and si-CCDC183-AS1#1 + TCF7L2 vector. (c) Glucose uptake was determined in UMUC3 or SW780 cells preinfected with si-NC, si-CCDC183-AS1#1, and si-CCDC183-AS1#1 + TCF7L2 vector. (d) Lactate production in UMUC3 or SW780 cells preinfected with si-NC, si-CCDC183-AS1#1, and si-CCDC183-AS1#1 + TCF7L2 vector. (e) ATP level was determined in UMUC3 or SW780 cells preinfected with si-NC, si-CCDC183-AS1#1, and si-CCDC183-AS1#1 + TCF7L2 vector.

Subsequently, it was revealed that TCF7L2 overexpression has an opposite effect to CCDC183-AS1 knockdown on ECAR (Figure 8(a)), OCR (Figure 8(b)), glucose uptake (Figure 8(c)), lactate production (Figure 8(d)), and ATP level (Figure 8(e)) in UMUC3 and SW780 cells, confirming that CCDC183-AS1 modulates aerobic glycolysis in BC via upregulating TCF7L2.

### 3.8. MITF Transcriptionally Regulates CCDC183-As1 Expression in BC Cell

**Figure 9:**
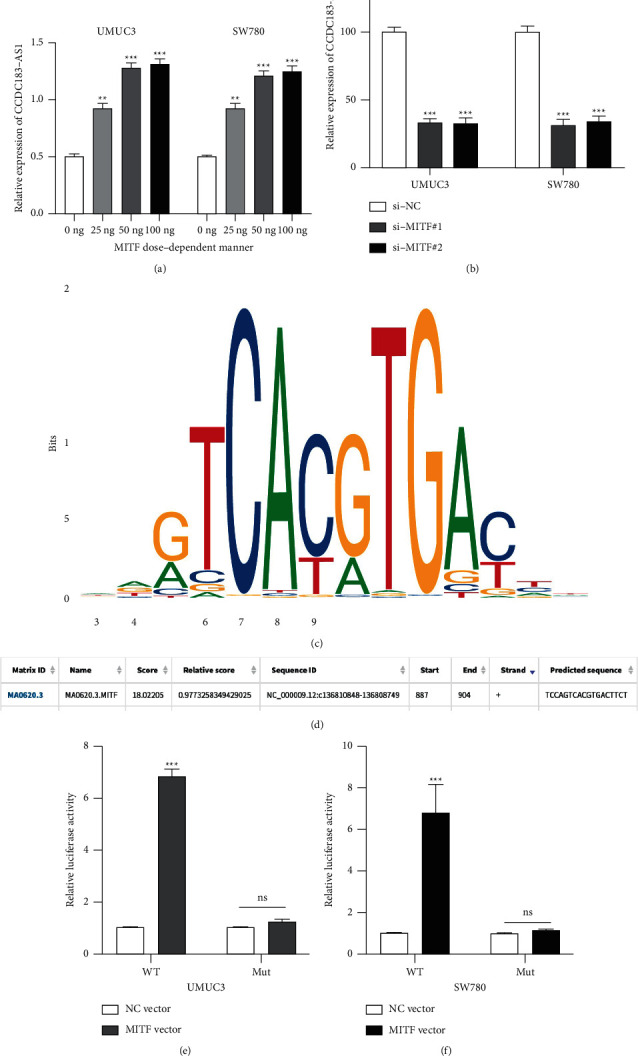
MITF transcriptionally regulates CCDC183-AS1 expression in BC cells. (a) CCDC183-AS1 expression in UMUC3 and SW780 cells on 0/20/50/100 ng MITF transfection was measured by RT-PCR. (b) CCDC183-AS1 expression in UMUC3 and SW780 cells preinfected with si-NC, si-MITF#1, and si-MITF#2 was measured by RT-PCR. (c) Predicted binding sites on MITF by the JASPAR database. (d) Predicted binding sites of CCDC183-AS1 promoter on MITF by the JASPAR database. (e, f) The dual-luciferase assay was conducted to determine the association between MITF on the CCDC183-AS1 promoter.

Accumulating evidence suggests that some translational factors may contribute to the dysregulation of lncRNAs [19,20]. Hereby, by utilizing the JASPAR database, it was found that Melanocyte Inducing Transcription Factor (MITF) might transcriptionally regulate CCDC183-AS1 expression. Subsequently, it was revealed that the CCDC183-AS1 level was positively modulated by MITF in a dose-dependent manner (Figure 9(a)). Furthermore, CCDC183-AS1 expression decreased in MITF downregulated UMUC3 and SW780 cells (Figure 9(b)). These data suggested that MITF regulates CCDC183-AS1. To validate that MITF is a transcription factor of CCDC183-AS1, the predicted binding sites between MITF (Figure 9(c)) and CCDC183-AS1 (Figure 9(d)) were obtained from JASPAR. Results of the dual-luciferase reporter gene assay suggested that MITF directly targets the promoter of CCDC183-AS1 in UMUC3 and SW780 cells (Figures 9(e) and 9(f)). Our results revealed that CCDC183-AS1 expression could be transcriptionally activated by MITF.

## 4. Discussion

BC is the primary malignant tumor of the genitourinary tract and a huge healthcare burden [21]. BC patients with an advanced stage or chemo-resistance phenomena have a poor prognosis and outcomes [22,23]. Due to the complex process and epigenetic abnormalities of BC, the molecular mechanisms behind BC development have been studied in-depth in the past decades, such as modifications of DNA and histone, chromatin remodeling, RNA methylation, noncoding RNAs, and ubiquitination [24–30]. Emerging evidence suggests that lncRNA is a hot topic in recent years [28, 31, 32]. Our study identified a novel functional lncRNA CCDC183-AS1, which plays an oncogene role in BC progression.

It has been well documented that lncRNA acts as a molecular sponge for microRNAs and transcriptionally regulates gene expression. Indeed, a lncRNA/miRNA/mRNA network has been studied in various diseases, especially in cancer [6, 33–37]. Herein, our study investigated the downstream mechanisms of CCDC183-AS1 in BC and identified the potential miRNA/mRNA axis, the novel CCDC183-AS1/miR-4731-5p/TCF7L2 axis. Furthermore, studies have confirmed that transcription factors can regulate lncRNA expression in multiple cell types [38–41]. By conducting bioinformatics analysis and luciferase reporter assays, our results suggest that MITF transcriptionally regulates CCDC183-AS1 expression in BC cells. MITF belongs to the helix-loop-helix leucine zipper (b-HLH-zip) family, the functions of which have been investigated in-depth in the development and maintenance of melanoma. The dysregulation of MITF is involved in cellular behaviors including proliferation, migration, and invasion [42–46]. Notably, none of them has been well studied in BC, and our results demonstrated the biological functions and tumor expression of each gene in BC progression, which enriched the research profiles for the MITF/CCDC183-AS1/miR-4731-5p/TCF7L2 axis.

TCF7L2 is an essential gene in the modulation of aerobic glycolysis (the Warburg effect) [17, 18, 47]. Aerobic glycolysis has a crucial role in tumor progression, maintenance, and cell transformation [48–50], so our study investigated whether CCDC183-AS1 exerts its function on aerobic glycolysis in BC cells. Our results suggest that CCDC183-AS1 positively regulated aerobic glycolysis in BC cells by regulating TCF7L2, which proved a new insight into the study of aerobic glycolysis in BC.

Although our results have partially demonstrated the molecular relationships among the MITF/CCDC183-AS1/miR-4731-5p/TCF7L2 axis and revealed the biological roles of the axis in BC development, the clinical significance of each gene in BC needs large-scale human samples and data for further investigation. Also, the in vitro experiment results need to be further confirmed in vivo.

In conclusion, our results demonstrated that CCDC183-AS1 functions as an oncogene in BC progression. CCDC183-AS1 knockdown suppressed cell proliferation, migration, invasion, and aerobic glycolysis levels. The novel MITF/CCDC183-AS1/miR-4731-5p/TCF7L2 axis identified in BC may be a promising diagnostic or treatment target for BC in the future.

## Data Availability

The datasets supporting the conclusions of this article can be obtained from the corresponding author under reasonable request.

## Abbreviations

BC: Bladder cancer
MTIF: Melanocyte-inducing transcription factor
lncRNA: Long noncoding RNA
CCDC183-AS1: Coiled-coil domain-containing 183 antisense RNA 1
TCF7L2: Transcription factor 7 like 2
CCK-8: Cell count kit-8
EdU: 5-Ethynyl-2′-deoxyuridine
ECAR: Extracellular acidification rate
OCR: Oxygen consumption rate
WT: Wide type
MUT: Mutant type.

## References

[1] Bray F., Ferlay J., Soerjomataram I., Siegel R. L., Torre L. A., Jemal A. (2018). Global cancer statistics 2018: GLOBOCAN estimates of incidence and mortality worldwide for 36 cancers in 185 countries. *A Cancer Journal for Clinicians*.

[2] Alfred Witjes J., Lebret T., Compérat E. M. (2017). Updated 2016 EAU guidelines on muscle-invasive and metastatic bladder cancer. *European Urology*.

[3] Antoni S., Ferlay J., Soerjomataram I., Znaor A., Jemal A., Bray F. (2017). Bladder cancer incidence and mortality: a global overview and recent trends. *European Urology*.

[4] Carlevaro-Fita J., Johnson R. (2019). Global positioning system: understanding long noncoding RNAs through subcellular localization. *Molecular Cell*.

[5] Lin C., Yang L. (2018). Long noncoding RNA in cancer: wiring signaling circuitry. *Trends in Cell Biology*.

[6] Huang Y. (2018). The novel regulatory role of lncRNA-miRNA-mRNA axis in cardiovascular diseases. *Journal of Cellular and Molecular Medicine*.

[7] Wu S., Bono J., Tao Y. X. (2019). Long noncoding RNA (lncRNA): a target in neuropathic pain. *Expert Opinion on Therapeutic Targets*.

[8] Feng S. D., Yang J. H., Yao C. H. (2017). Potential regulatory mechanisms of lncRNA in diabetes and its complications. *Biochemistry and Cell Biology*.

[9] Bhan A., Soleimani M., Mandal S. S. (2017). Long noncoding RNA and cancer: a new Paradigm. *Cancer Research*.

[10] Chen C., Luo Y., He W. (2019). Exosomal long noncoding RNA LNMAT2 promotes lymphatic metastasis in bladder cancer. *Journal of Clinical Investigation*.

[11] He W., Zhong G., Jiang N. (2018). Long noncoding RNA BLACAT2 promotes bladder cancer-associated lymphangiogenesis and lymphatic metastasis. *Journal of Clinical Investigation*.

[12] Luo W., Wang J., Xu W. (2021). LncRNA RP11-89 facilitates tumorigenesis and ferroptosis resistance through PROM2-activated iron export by sponging miR-129-5p in bladder cancer. *Cell Death and Disease*.

[13] Tan J., Liu B., Zhou L. (2022). LncRNA TUG1 promotes bladder cancer malignant behaviors by regulating the miR-320a/FOXQ1 axis. *Cellular Signalling*.

[14] Zhao S. J., Shen Y. F., Li Q. (2018). SLIT2/ROBO1 axis contributes to the Warburg effect in osteosarcoma through activation of SRC/ERK/c-MYC/PFKFB2 pathway. *Cell Death and Disease*.

[15] Zhao Z., Sun W., Guo Z., Zhang J., Yu H., Liu B. (2020). Mechanisms of lncRNA/microRNA interactions in angiogenesis. *Life Sciences*.

[16] Li J. H., Liu S., Zhou H., Qu L. H., Yang J. H. (2014). Starbase v2.0: decoding miRNA-ceRNA, miRNA-ncRNA and protein-RNA interaction networks from large-scale CLIP-Seq data. *Nucleic Acids Research*.

[17] Malakar P., Stein I., Saragovi A. (2019). Long noncoding RNA MALAT1 regulates cancer glucose metabolism by enhancing mTOR-mediated translation of TCF7L2. *Cancer Research*.

[18] Xiang J., Hu Q., Qin Y. (2018). TCF7L2 positively regulates aerobic glycolysis via the EGLN2/HIF-1*α* axis and indicates prognosis in pancreatic cancer. *Cell Death and Disease*.

[19] Dykes I. M., Emanueli C. (2017). Transcriptional and Post-transcriptional gene regulation by long non-coding RNA. *Genomics, Proteomics and Bioinformatics*.

[20] Sarkar D., Leung E. Y., Baguley B. C., Finlay G. J., Askarian-Amiri M. E. (2015). Epigenetic regulation in human melanoma: past and future. *Epigenetics*.

[21] Kurtova A. V., Xiao J., Mo Q. (2015). Blocking PGE2-induced tumour repopulation abrogates bladder cancer chemoresistance. *Nature*.

[22] Porten S. P. (2018). Epigenetic alterations in bladder cancer. *Current Urology Reports*.

[23] Audenet F., Attalla K., Sfakianos J. P. (2018). The evolution of bladder cancer genomics: what have we learned and how can we use it?. *Urologic Oncology: Seminars and Original Investigations*.

[24] Zhang T., Du E., Liu Y. (2020). Anticancer effects of zinc Oxide nanoparticles through altering the methylation status of histone on bladder cancer cells. *International Journal of Nanomedicine*.

[25] Lee M., Kim B., Kim V. N. (2014). Emerging roles of RNA modification: m6A and U-tail. *Cell*.

[26] Geula S., Moshitch-Moshkovitz S., Dominissini D. (2015). m 6 A mRNA methylation facilitates resolution of naïve pluripotency toward differentiation. *Science*.

[27] Yang X., Ye T., Liu H. (2021). Expression profiles, biological functions and clinical significance of circRNAs in bladder cancer. *Molecular Cancer*.

[28] Li Y., Li G., Guo X., Yao H., Wang G., Li C. (2020). Non-coding RNA in bladder cancer. *Cancer Letters*.

[29] Masclef L., Ahmed O., Estavoyer B. (2021). Roles and mechanisms of BAP1 deubiquitinase in tumor suppression. *Cell Death and Differentiation*.

[30] Sanli O., Dobruch J., Knowles M. A. (2017). Bladder cancer. *Nature Reviews Disease Primers*.

[31] Quan J., Pan X., Zhao L. (2018). LncRNA as a diagnostic and prognostic biomarker in bladder cancer: a systematic review and meta-analysis. *OncoTargets and Therapy*.

[32] Cao Y., Tian T., Li W. (2020). Long non-coding RNA in bladder cancer. *Clinica Chimica Acta*.

[33] Ma N., Tie C., Yu B., Zhang W., Wan J. (2020). Identifying lncRNA-miRNA-mRNA networks to investigate Alzheimer’s disease pathogenesis and therapy strategy. *Aging*.

[34] Zhu J., Zhang X., Gao W., Hu H., Wang X., Hao D. (2019). lncRNA/circRNA‑miRNA‑mRNA ceRNA network in lumbar intervertebral disc degeneration. *Molecular Medicine Reports*.

[35] Goodall G. J., Wickramasinghe V. O. (2021). RNA in cancer. *Nature Reviews Cancer*.

[36] Han T. S., Hur K., Cho H. S., Ban H. S. (2020). Epigenetic associations between lncRNA/circRNA and miRNA in hepatocellular carcinoma. *Cancers*.

[37] Wang J. Y., Yang Y., Ma Y. (2020). Potential regulatory role of lncRNA-miRNA-mRNA axis in osteosarcoma. *Biomedicine and Pharmacotherapy*.

[38] Wang J., Gu J., You A. (2020). The transcription factor USF1 promotes glioma cell invasion and migration by activating lncRNA HAS2-AS1. *Bioscience Reports*.

[39] Zhao X., Chen L., Wu J., You J., Hong Q., Ye F. (2021). Transcription factor KLF15 inhibits the proliferation and migration of gastric cancer cells via regulating the TFAP2A-AS1/NISCH axis. *Biology Direct*.

[40] Gu Z., Zhou Y., Cao C., Wang X., Wu L., Ye Z. (2020). TFAP2C-mediated LINC00922 signaling underpins doxorubicin-resistant osteosarcoma. *Biomedicine & Pharmacotherapy*.

[41] Fan H., Yuan J., Li Y. (2021). MKL1-induced lncRNA SNHG18 drives the growth and metastasis of non-small cell lung cancer via the miR-211-5p/BRD4 axis. *Cell Death and Disease*.

[42] Hodgkinson C. A., Moore K. J., Nakayama A. (1993). Mutations at the mouse microphthalmia locus are associated with defects in a gene encoding a novel basic-helix-loop-helix-zipper protein. *Cell*.

[43] Goding C. R., Arnheiter H. (2019). MITF-the first 25 years. *Genes and Development*.

[44] Cancer Genome Atlas Network (2015). Genomic classification of cutaneous melanoma. *Cell*.

[45] Cheli Y., Ohanna M., Ballotti R., Bertolotto C. (2010). Fifteen-year quest for microphthalmia-associated transcription factor target genes. *Pigment Cell and Melanoma Research*.

[46] Hoek K. S., Schlegel N. C., Eichhoff O. M. (2008). Novel MITF targets identified using a two-step DNA microarray strategy. *Pigment Cell and Melanoma Research*.

[47] Zhao Z., Wang L., Bartom E. (2019). *β*-Catenin/Tcf7l2-dependent transcriptional regulation of GLUT1 gene expression by Zic family proteins in colon cancer. *Science Advances*.

[48] Fantin V. R., St-Pierre J., Leder P. (2006). Attenuation of LDH-A expression uncovers a link between glycolysis, mitochondrial physiology, and tumor maintenance. *Cancer Cell*.

[49] Le A., Cooper C. R., Gouw A. M. (2010). Inhibition of lactate dehydrogenase A induces oxidative stress and inhibits tumor progression. *Proceedings of the National Academy of Sciences*.

[50] Patra K. C., Wang Q., Bhaskar P. T. (2013). Hexokinase 2 is required for tumor initiation and maintenance and its systemic deletion is therapeutic in mouse models of cancer. *Cancer Cell*.

